# Ferroelectric polarization and magnetic structure at domain walls in a multiferroic film

**DOI:** 10.1038/s41467-024-50431-9

**Published:** 2024-07-19

**Authors:** Ang Tao, Yixiao Jiang, Shanshan Chen, Yuqiao Zhang, Yi Cao, Tingting Yao, Chunlin Chen, Hengqiang Ye, Xiu-Liang Ma

**Affiliations:** 1 Shenyang National Laboratory for Materials Science, Institute of Metal Research, Chinese Academy of Sciences, 110016 Shenyang, China; 2 School of Materials Science and Engineering, University of Science and Technology of China, 110016 Shenyang, China; 3 Jihua Lab, 528251 Foshan, China; 4 Institute of Quantum and Sustainable Technology (IQST), School of Chemistry and Chemical Engineering, Jiangsu University, 212013 Zhenjiang, Jiangsu China; 5 Foshan (Southern China) Institute for New Materials, 528200 Foshan, Guangdong China; 6 Bay Area Center for Electron Microscopy, Songshan Lake Materials Laboratory, 523808 Dongguan, Guangdong China; 7 Institute of Physics, Chinese Academy of Sciences, 100190 Beijing, China

**Keywords:** Ferroelectrics and multiferroics, Transmission electron microscopy, Surfaces, interfaces and thin films

## Abstract

Domain walls affect significantly ferroelectric and magnetic properties of magnetoelectric multiferroics. The stereotype is that the ferroelectric polarization will reduce at the domain walls due to the incomplete shielding of depolarization field or the effects of gradient energy. By combining transmission electron microscopy and first-principles calculations, we demonstrate that the ferroelectric polarization of tail-to-tail 180° domain walls in ε-Fe_2_O_3_ is regulated by the bound charge density. A huge enhancement (43%) of ferroelectric polarization is observed in the type I domain wall with a low bound charge density, while the ferroelectric polarization is reduced to almost zero at the type II domain wall with a high bound charge density. The magnetic coupling across the type I and type II ferroelectric domain walls are antiferromagnetic and ferromagnetic, respectively. Revealing mechanisms for enhancing ferroelectric polarization and magnetic behaviors at ferroelectric domain walls may promote the fundamental research and potential applications of magnetoelectric multiferroics.

## Abstract (web summary)

The ferroelectric polarization generally reduces at the domain walls. Here, the authors demonstrate that a huge enhancement (43%) of ferroelectric polarization in ε-Fe_2_O_3_ in the domain wall with a low bound charge density.

## Introduction

Multiferroic materials, especially magnetoelectric multiferroics exhibiting both ferromagnetic and ferroelectric properties in the same phase, have recently attracted intense interest due to their great potential for microelectronic storage applications^1–6^. In principle, magnetoelectric multiferroics must contain simultaneously both ferroelectric and magnetic domain walls (DWs) due to the co-existence of ferroelectric and spin polarizations in a material.

Many efforts have been devoted to the investigation of ferroelectric DWs in magnetoelectric multiferroics due to their abundant and fascinating physical phenomena, such as the electrical conductivity^7–9^, photovoltaic response^10^, magnetoresistvity^11^, and spin transport^12^. According to the presence of bound charges or not, ferroelectric DWs are divided into neutral (head-to-tail or antiparallel) and charged (head-to-head or tail-to-tail) ones. In general, the spontaneous polarization tends to decrease at either neutral or charged ferroelectric domain wall though the intrinsic mechanisms are different. The polarization reduces at the neutral DWs due to the effects of the gradient energy^13,14^. For the charged DWs, free carriers cannot completely shield the depolarization field generated by bound charges^15–19^, thereby leading to the reduction of the ferroelectric polarization. The phenomenon of reduced polarization near the DWs has been found in many ferroelectrics and multiferroics, such as PbZr_0.2_Ti_0.8_O_3_^17^, BiFeO_3_^18,19^, and YMnO_3_^20^. Whether the spontaneous polarization at ferroelectric DWs can be increased or not is a fascinating issue of great scientific and practical significance.

For magnetoelectric multiferroic materials, the ferroelectric DWs could play an important role in modulating the magnetic properties of materials. Revealing the magnetic coupling nature at the ferroelectric DWs is of critical importance for in-depth understanding of the magnetoelectric behaviors of magnetoelectric multiferroics. ε-Fe_2_O_3_ is a promising room-temperature binary magnetoelectric multiferroic of a huge magnetic coercivity of ~20 kOe^21^, magnetoelectric coupling phenomenon^22^, and discriminable domain walls^23,24^, rendering it be a good model material for the investigation of ferroelectric and magnetic behaviors at DWs.

In this work, we systematically investigate the atomic and electronic structures, ferroelectric and magnetic properties of DWs in ε-Fe_2_O_3_ by combining aberration-corrected transmission electron microscopy and spin-polarized first-principles calculations. It is demonstrated that the ferroelectric polarization at ε-Fe_2_O_3_ DWs depends on their atomic structures. The ferroelectric polarization at the type I ferroelectric DW has a huge enhancement of ~43%, while that at the type II DW is reduced to almost zero. The type I and type II ferroelectric DWs exhibit the antiferromagnetic and ferromagnetic magnetic couplings, respectively.

## Results

Supplementary Fig. 1 shows the microstructure of as-prepared ε-Fe_2_O_3_ thin film obtained through the ion-irradiation-induced α to ε phase transformation. The α-Fe_2_O_3_ epitaxial thin film on the SrTiO_3_ (111) substrate is single-crystalline before phase transformation. After phase transformation, the ε-Fe_2_O_3_ film is polycrystalline and has a high purity, which are similar as those fabricated by pulsed laser deposition (PLD)^24^ and chemical vapor deposition^25^. There is no remaining α-Fe_2_O_3_ in the ε-Fe_2_O_3_ thin film. Two types of orientation relationships between film and substrate are formed: [100]_ε_//$[11\bar{2}]$_sub_, [001]_ε_//$[111]$_sub_ and $[\bar{1}10]$_ε_//$[11\bar{2}]$_sub_, [001]_ε_//$[111]$_sub_, respectively. Thus, the atomic structure of DWs in ε-Fe_2_O_3_ are determined along the [100] and $[\bar{1}10]$ zone axes, which facilitates us to establish the 3D atomic configuration of DWs. Basically, two types of tail-to-tail 180° DWs have been identified in the ε-Fe_2_O_3_ film.

**Fig. 1:**
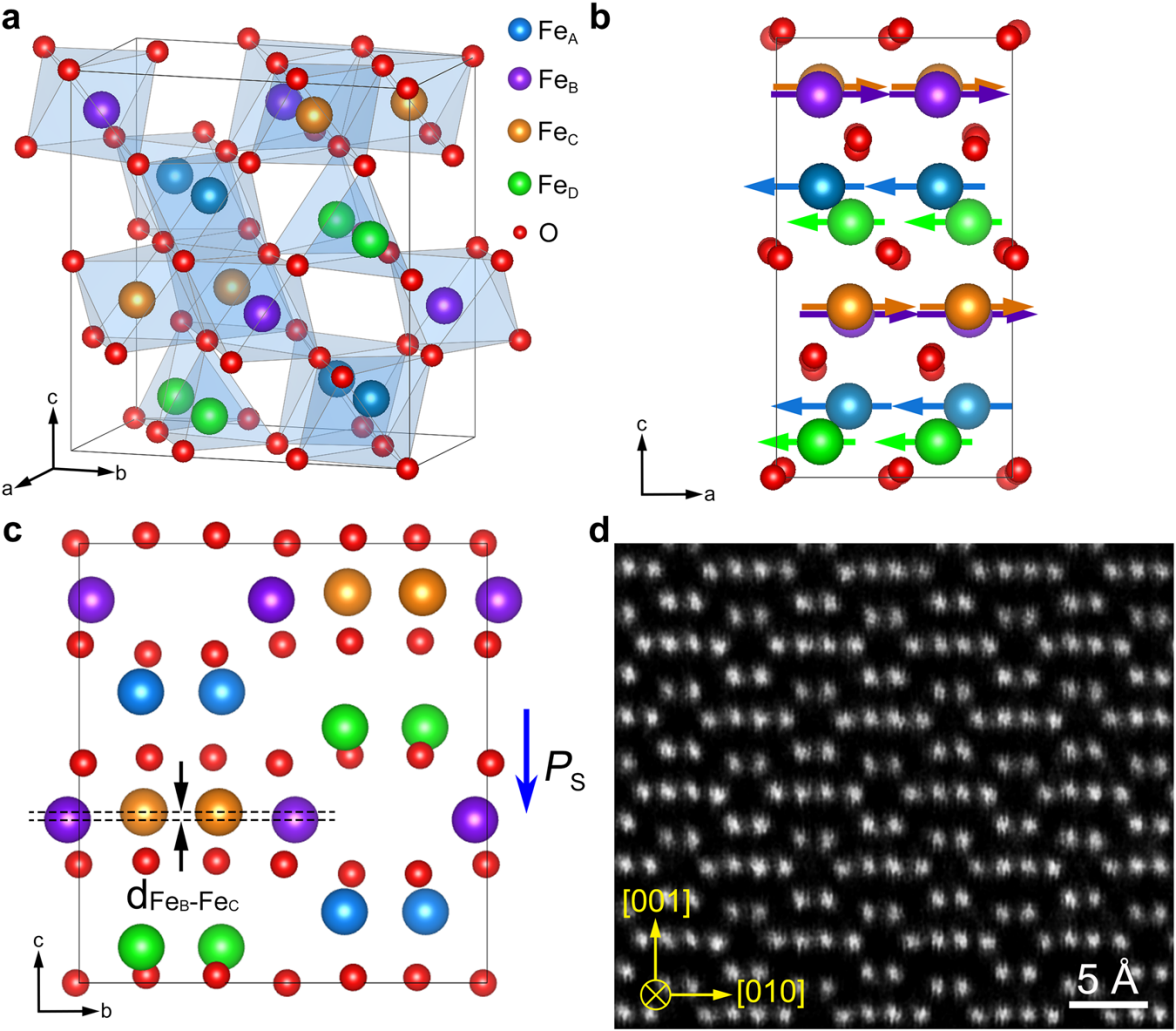
Schematic diagrams illustrating the atomic structure, magnetic structure, and ferroelectric polarization of bulk ε-Fe_2_O_3_. **a** Atomic model of the ε-Fe_2_O_3_ unit cell. Fe_A_, Fe_B_, and Fe_C_ atoms locate in the center of O octahedra, while Fe_D_ atoms in the O tetrahedra. **b** The magnetic structure of ε-Fe_2_O_3_ viewed along the [010] direction. **c** Schematic diagram revealing the structural origin for the ferroelectric polarization of ε-Fe_2_O_3_. The ferroelectric polarization is along the [$00\bar{1}$] axis, and the magnitude is positively correlated with d_FeB-FeC_. **d** HAADF-STEM image of ε-Fe_2_O_3_ along the [$\bar{1}00$] direction.

Figure 1 shows the schematic diagrams illustrating the atomic structure, magnetic structure, and ferroelectric polarization of bulk ε-Fe_2_O_3_. Figure 1a is the 3D atomic model of ε-Fe_2_O_3_, which has an orthorhombic unit cell (*a* = 5.095 Å, *b* = 8.789 Å, *c* = 9.437 Å) with the Pna2_1_ space group^26^. Fe atoms in ε-Fe_2_O_3_ have four different crystallographic sites: Fe_A_, Fe_B_, and Fe_C_ atoms locate in the center of O octahedra, while Fe_D_ atoms in the O tetrahedra. The magnetic structure of ε-Fe_2_O_3_ is presented in Fig. 1b viewed along the [010] direction. The magnetic moments of Fe_A_ and Fe_D_ atoms are along the [$\bar{1}00$] direction, while those of Fe_B_ and Fe_C_ atoms along the [100] direction. The magnetic moments of Fe_D_ atoms are smaller than those of Fe_A_, Fe_B_, and Fe_C_ atoms^27,28^. As shown in Fig. 1c, the direction of the ferroelectric polarization of ε-Fe_2_O_3_ is along the $[00\bar{1}]$ axis (more details are shown in note 1). The Fe_B_ and Fe_C_ atomic planes have a displacement along the c-axis. The magnitude of ferroelectric polarization is positively correlated with the height difference (d_FeB-FeC_) between the Fe_B_ and Fe_C_ atomic planes^23^. The magnetic and ferroelectric polarizations have the orthogonal directions, thereby maintaining the stability of the multiferroic structure. Figure 1d is a typical high-angle annular dark-filed (HAADF) image of the bulk ε-Fe_2_O_3_ along the [$\bar{1}00$] direction. It is clear that there is a height difference between the Fe_B_ and Fe_C_ atomic planes, indicating the existence of the ferroelectric polarization. (The typical HAADF image of the bulk ε-Fe_2_O_3_ along the $[\bar{1}10]$ direction is shown in Supplementary Fig. 2). Since the ε-Fe_2_O_3_ TEM samples obtained by irradiation-induced phase transformation are too small to be used for the experimental measurement of ferroelectric and ferromagnetic properties, ε-Fe_2_O_3_ thin films were grown directly on Nb doped SrTiO_3_ (111) substrates using the PLD technique (more details in Method section). Bright-field TEM image, SAED pattern and XRD pattern of the PLD-deposited ε-Fe_2_O_3_ thin film are shown in Supplementary Fig. 3a–c. It is clear that the PLD-deposited ε-Fe_2_O_3_ has a very similar microstructure as the irradiation-induced ε-Fe_2_O_3_, especially in the SAED patterns. Ferroelectric and magnetic performance of the PLD-deposited ε-Fe_2_O_3_ thin films are shown in Supplementary Fig. 3d–h. All the magnetization hysteresis loop, ferroelectric hysteresis loop, amplitude and phase PFM loops, phase and amplitude images suggest that the ε-Fe_2_O_3_ thin films are ferroelectric and ferromagnetic at room temperature.

**Fig. 2:**
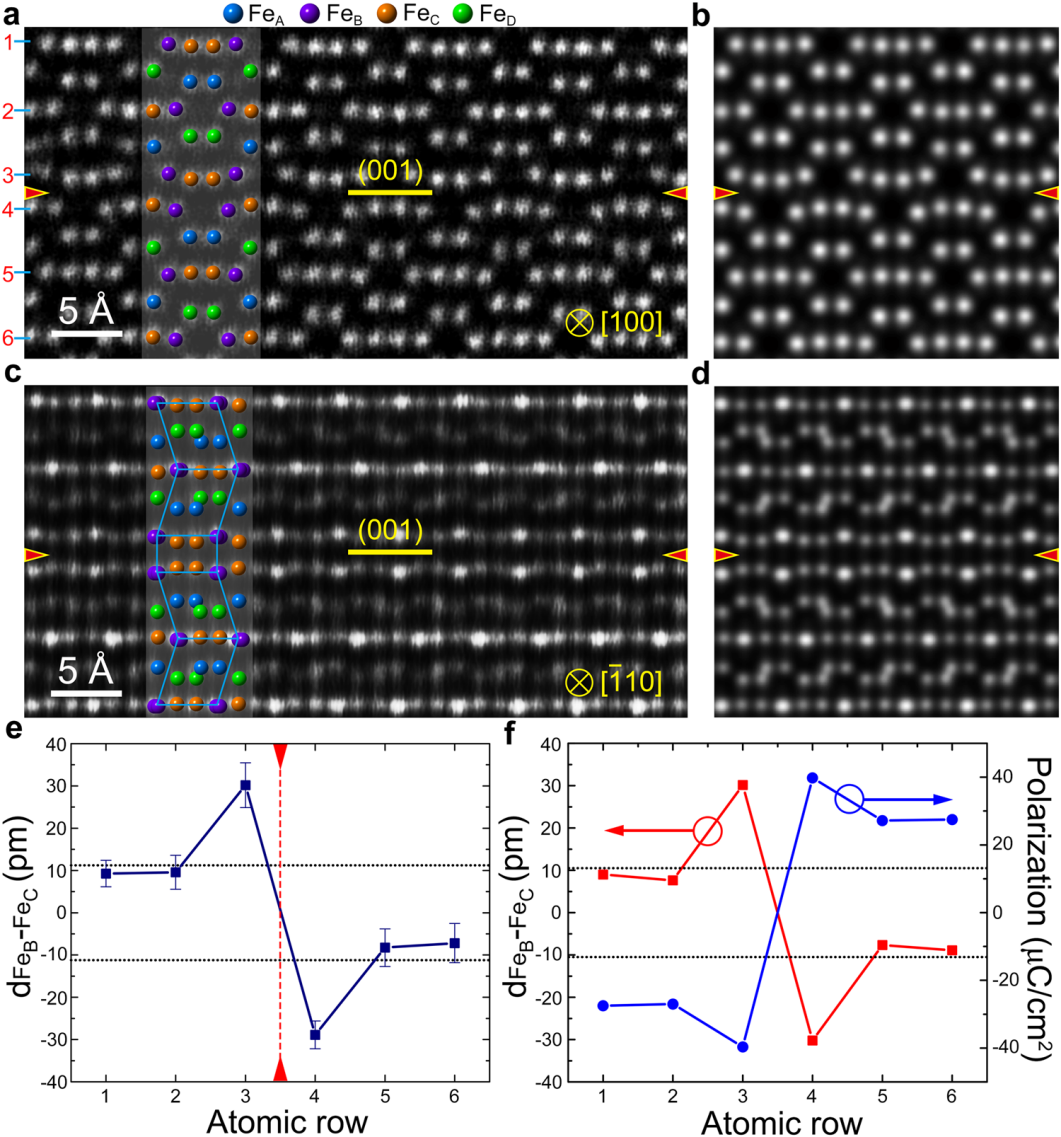
Atomic structures and ferroelectric polarization of the ε-Fe_2_O_3_ type I DW. **a**, **c** HAADF-STEM images of the type I DW along the [100] and [$\bar{1}10$] direction, respectively. The DWs are denoted by red arrows. Atomic models are inserted to demonstrate clearly the atomic structure of DWs. The d_FeB-FeC_ is significantly increased near the DW. **b**, **d** Corresponding simulated HAADF-STEM images. **e** Quantitative analysis of the d_FeB-FeC_ values across the type I DW. The error bar is the standard deviation. The DW is marked by the red dashed line. The black dotted lines indicate the d_FeB-FeC_ in the bulk ε-Fe_2_O_3_. **f** Calculated d_FeB-FeC_ and ferroelectric polarization near the DW. The ferroelectric polarization near the DW is significantly increased.

HAADF observations revealed that there are two types of the tail-to-tail 180° DWs formed in both the irradiation-induced and PLD-deposited ε-Fe_2_O_3_ thin films. From the ε-Fe_2_O_3_ grains along the [100] and $[\bar{1}10]$ directions, the DWs have been imaged from two directions to obtain three-dimensional atomic structure information. Figure 2a–d present the experimental and simulated atomic-resolution HAADF images of the type I DW along the [100] and $[\bar{1}10]$ directions in the irradiation-induced ε-Fe_2_O_3_ thin film, respectively. The corresponding ABF of type I DW along the [100] is shown in Supplementary Fig. 4b. The DWs are denoted by red arrows. Atomic models are inserted to demonstrate clearly the atomic structure of DWs. As shown in Fig. 2a, c, the type I DW is a tail-to-tail 180° DW since the ferroelectric polarization reverse the direction across the DW. Interestingly, the height difference (d_FeB-FeC_) between the Fe_B_ and Fe_C_ atomic planes is significantly increased near the DW, suggesting that the ferroelectric polarization near the DW should be larger than that of the bulk. A quantitative analysis of the d_FeB-FeC_ values across the type I DW is performed based on the HAADF image in Fig. 2a by using the CalAtom Software^29^ (Detailed procedures are shown in Supplementary Fig. 5). The average d_FeB-FeC_ of each atomic planes are shown in Fig. 2e. The atomic planes adjacent with the DW have much larger d_FeB-FeC_ values (i.e., 30.2 pm and −28.9 pm), while the d_FeB-FeC_ values in the bulk are ±11.4 pm (as indicted by the dashed lines). Based on the HAADF images in Fig. 2a, c, first-principles calculations have been carried out to reveal the atomic structure and ferroelectric properties of the DW. Using the atomic models after structural relaxation, HAADF images of the type I DW are simulated along the [100] and $[\bar{1}10]$ directions, and the results are shown in Fig. 2b, d. It is clear that the simulated HAADF images are consistent well with the experimental counterparts. The theoretical d_FeB-FeC_ values obtained from Fig. 2b are presented by the red line in Fig. 2f, matching well with the experimental ones (i.e., Fig. 2e). These facts indicate that the atomic models for first-principles calculations are correct. The ferroelectric polarization near the DW is calculated using the Born effective charges method. As denoted by the blue line in Fig. 2f, the ferroelectric polarization is positively correlated to d_FeB-FeC_ in magnitude but opposite in direction. The ferroelectric polarization near the DW is ~40 μC/cm^2^, which is 43% larger than that of the bulk (i.e., 28 μC/cm^2^).

**Fig. 3:**
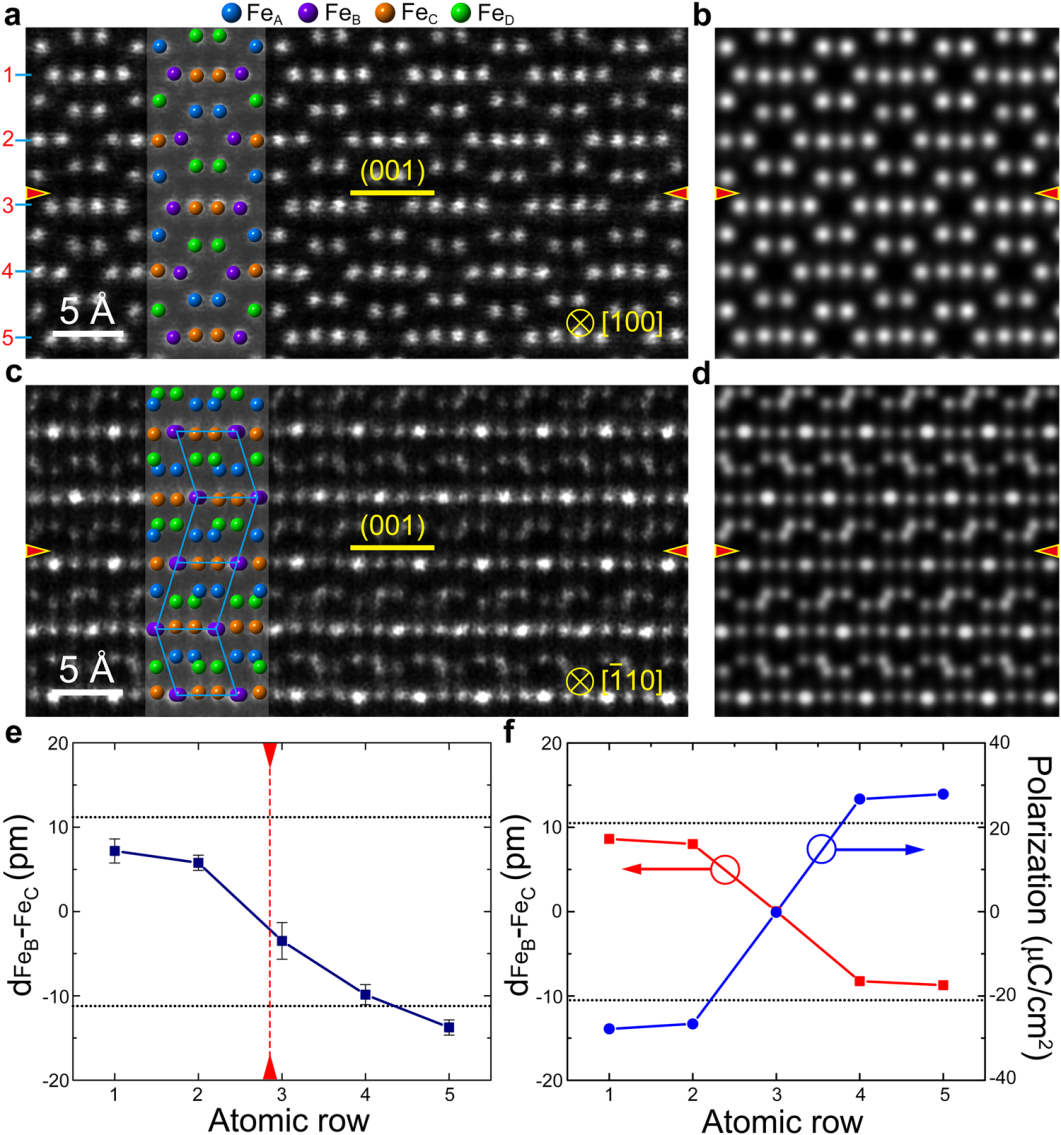
Atomic structures and ferroelectric polarization of the type II DW. **a**, **c** HAADF-STEM images of the type II DW along the [100] and [$\bar{1}10$] direction, respectively. The d_FeB-FeC_ is significantly decreased near the DW. **b**, **d** Corresponding simulated HAADF-STEM images. The DWs are indicated by red arrows**. e** Quantitative analysis of the d_FeB-FeC_ values across the type II DW. The DW is denoted by the red dashed line. The black dotted lines indicate the d_FeB-FeC_ in the bulk ε-Fe_2_O_3_. **f** Calculated d_FeB-FeC_ and ferroelectric polarization near the DW. The ferroelectric polarization near the DW is reduced to almost zero.

The atom-resolved experimental and simulated HAADF images of the type II DW are shown in Fig. 3a–d. The corresponding ABF of type II DW along the [100] is shown in Supplementary Fig. 4d.The DWs are denoted by red arrows. The type II DW is also a tail-to-tail 180° DW. In contrast to the type I DW, the d_FeB-FeC_ values near the type II DW are lower than that in the bulk, suggesting that the ferroelectric polarization near the type II DW is reduced. The experimentally measured d_FeB-FeC_ values based on Fig. 3a are shown in Fig. 3e. It is clear that the averaged d_FeB-FeC_ value decreases to almost zero at the type II DW, suggesting that the ferroelectric polarization near the DW is significantly reduced. The simulated HAADF images in Fig. 3b, d, and the theoretically calculated d_FeB-FeC_ curve in Fig. 3f match well with the experimental counterparts. As denoted by the blue line in Fig. 3f, the ferroelectric polarization gradually decreases from 28 μC/cm^2^ in bulk to almost zero near the type II DW. The HAADF and ABF images of type I and type II DWs in the PLD-deposited ε-Fe_2_O_3_ thin films are shown in Supplementary Fig. 6. As one can see, the type I and type II DWs in the PLD-deposited ε-Fe_2_O_3_ thin films have the same atomic structures as those in the irradiation-induced ε-Fe_2_O_3_ thin films. Thus, the type I and type II DWs in the irradiation-induced and PLD-deposited ε-Fe_2_O_3_ thin films should have similar ferroelectric and magnetic properties. We totally checked 38 DWs in the samples. The numbers of the type I and II DWs were found to be 15 and 23, respectively. This shows that the proportion of the two types of DWs is roughly equal. Supplementary Fig. 7 potential energy distribution across the DWs reveals the two DWs are indeed tail-to-tail type. To determine the valence state of Fe ions at the domain walls, EELS spectra including Fe-L and O-K edges were obtained from the type I and type II DWs and the bulk ε-Fe_2_O_3_, as shown in Supplementary Fig. 8. It is clear that the Fe-L and O-K edges obtained from the domain walls and the bulk are very similar, which indicate that the valence states of Fe ions at the domain walls and the bulk ε-Fe_2_O_3_ are 3+. It is reasonable since the stoichiometric defects will not change the valence state, similar to the cases in Fe_3_O_4_ twin boundaries^30^.

To confirm that the type I and type II DWs are indeed ferroelectric DWs instead of inversion twin boundaries, weak-beam dark filed imaging^31^ and atomic-resolution HAADF imaging along different directions were carried out. As shown in Supplementary Fig. 9a–c, the contrasts in the weak-beam dark-filed images remain consistent on both sides of the DWs. In addition, Supplementary Fig. 9d–i show the atomic-resolution HAADF images of the type I and type II DWs along the [100], [310] and [$\bar{1}10$] projections. It is clear that the atomic arrangement on both sides of the DWs are similar except for their different ferroelectric polarization. No evidence of twinning has been observed. Therefore, the results of weak-beam dark filed imaging and atomic-resolution HAADF imaging support that the type I and type II DWs are ferroelectric DWs, instead of inversion twin boundaries.

**Fig. 4:**
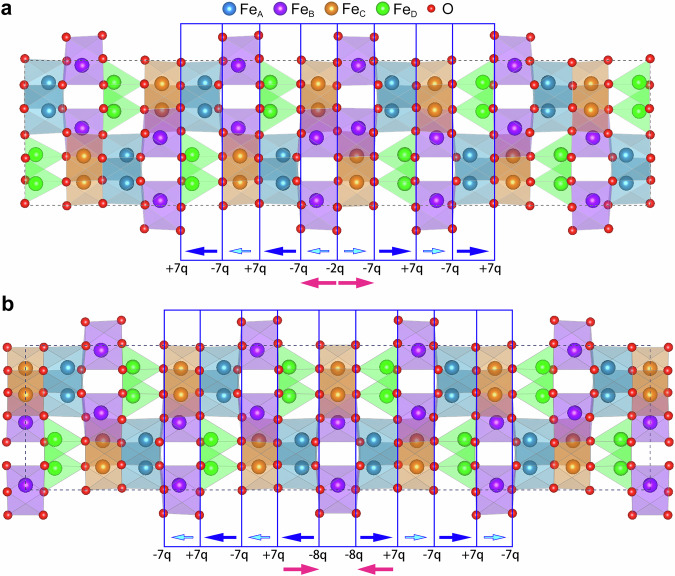
Schematics revealing mechanisms for the different trends of the ferroelectric polarization across the type I and II DWs. **a** Type I DW. **b** Type II DW. The spontaneous polarization of (Fe_A_Fe_D_)O_6_ layers is 8 times as that of (Fe_B_Fe_C_)O_6_ layers, which results in the bound charges of ±7q between the (Fe_B_Fe_C_)O_6_ and (Fe_A_Fe_D_)O_6_ layers. The bound charges of −2q and −8q are induced at the type I and type II DWs, respectively. The decrease of negative bound charges at the type I DW will induce a local electric field (Red arrows) pointing away from the DW in **a**, which will enhance the polarization. In contrast, a local electric field (Red arrows) pointing to the DW is formed due to the increase of negative bound charges at the type II DW in **b**, which will reduce the polarization.

Mechanisms for the different trends of the ferroelectric polarization across the type I and II DWs can be clarified in Fig. 4, which shows the spontaneous polarizations in different layers near the DWs. Due to the difference between the strength and orientation of Fe-O bonds in FeO_4_ tetrahedrons (Fe_D_) and FeO_6_ octahedrons (Fe_A_, Fe_B_, Fe_C_), the spontaneous polarization of (Fe_A_Fe_D_)O_6_ layers is 8 times as that of (Fe_B_Fe_C_)O_6_ layers. The significantly different magnitude of spontaneous polarization results in the bound charges of ±7q between the (Fe_B_Fe_C_)O_6_ and (Fe_A_Fe_D_)O_6_ layers, where q = |**P**|, where **P** means the spontaneous polarization in the (Fe_B_Fe_C_)O_6_ layer. The bound charges of −2q and −8q are induced at the type I and type II DWs, respectively. The decrease of negative bound charges at the type I DW will induce a local electric field pointing away from the DW (Fig. 4a), which will enhance the polarization. In contrast, a local electric field pointing to the DW is formed due to the increase of negative bound charges at the type II DW, which will reduce the ferroelectric polarization (Fig. 4b).

**Fig. 5:**
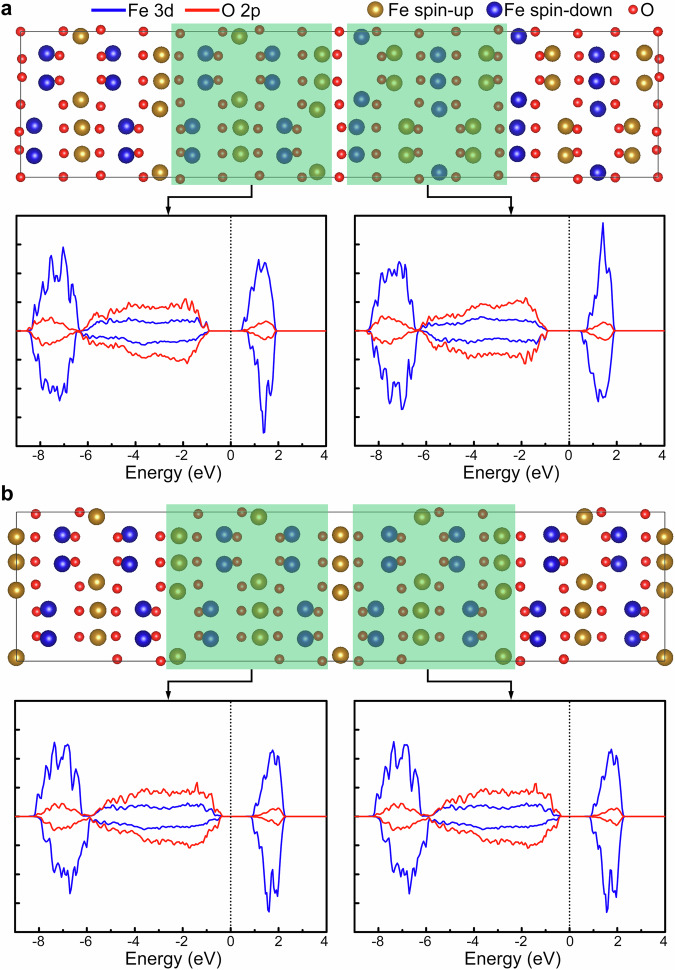
Spin-polarized local density of states (LDOS) across the type I and type II DWs. The E_F_ is denoted by the dashed lines. **a** LDOS across the type I DW. The net magnetic moments of the two conjugated domains for the type I DW are antiparallel, revealing the AFM coupling nature across the DW. **b** LDOS across the type II DW. The net magnetic moments of the two conjugated domains for the type II DW are parallel, indicating the FM coupling nature across the DW.

To clarify how the ferroelectric DWs affect the magnetic properties of ε-Fe_2_O_3_, the local magnetic couplings across the DWs are analyzed based on spin-polarized DFT calculations. The total ferromagnetic (FM) and antiferromagnetic (AFM) coupling between the two domains across the DWs are separately implemented in the calculations. For the type I DW, the formation energy of the AFM coupling is 0.38 J/m^2^, which is 0.20 J/m^2^ lower than that of the FM coupling, suggesting that the AFM coupling is more stable. Figure 5a shows the spin-polarized local density of states (LDOS) across the type I DW. The net magnetic moments of the two conjugated domains for the type I DW are antiparallel, revealing the AFM coupling nature across the DW. Thus the magnetic moments of the two conjugated domains would counteract each other, thereby reducing significantly the total spin polarization of ε-Fe_2_O_3_. For the type II DW, the formation energy of the FM coupling is 0.22 J/m^2^, which is 0.41 J/m^2^ lower than that of the AFM coupling, suggesting that the FM coupling is more stable than the AFM one. Figure 5b shows the spin-polarized LDOS across the type II DW. The net magnetic moments of the two conjugated domains for the type II DW are parallel, indicating the FM coupling nature across the DW. This fact suggests that the type II ferroelectric DW will not significantly affect the magnetic properties of ε-Fe_2_O_3_. More details for the magnetic coupling across the type I and type II DWs can be found in Supplementary Figs. 10 and 11. For reference, the LDOS of the bulk ε-Fe_2_O_3_ are shown in Supplementary Fig. 12.

In addition to the two types of tail-to-tail DWs observed experimentally, we computationally constructed two types of head-to-head DWs are energetically stable. Their ferroelectric and ferromagnetic analyses are shown in Supplementary Fig. 13 and Fig. 14. Compared with the tail-to-tail DWs, these two types of head-to-head DWs also show enhanced and decreased polarization, respectively. The ferromagnetic coupling modes across the DWs are also AFM and FM, respectively.

Clarifying how the DWs affect the ferroelectric and magnetic properties of magnetoelectric multiferroics is a fundamental issue of academic and practical significance. In this study, two types of tail-to-tail 180° DWs are found to be formed in ε-Fe_2_O_3_. The type I DW exhibit a huge enhancement (i.e., 43%) of ferroelectric polarization due to the low bound charge density, while the type II DW has a reduced ferroelectric polarization due to the high bound charge density. The magnetic coupling across the type I and type II ferroelectric DWs are antiferromagnetic and ferromagnetic, respectively. The findings that the ferroelectric polarization of DWs can be significantly increased and the ferroelectric DWs also play an important role in tailoring the magnetic properties of magnetoelectric multiferroics, will not only deepen our understanding of the abundant physical phenomena in magnetoelectric multiferroics and but also promote their practical applications.

## Methods

### Materials preparation and microscopic observations

The irradiation-induced ε-Fe_2_O_3_ films were obtained through an ion-radiation-induced α- to ε-Fe_2_O_3_ phase transformation. The fabrication of the α-Fe_2_O_3_ epitaxial thin films has been described in detail in the previous research^32^. TEM samples were fabricated by the Ar ion-milling process using a precision ion polishing system (Gatan 695) with a pressure of 10^−3 ^Pa at room temperature. Voltages of 0.1 − 4 kV were used to fabricate α-Fe_2_O_3_ TEM samples. The microstructure of the α-Fe_2_O_3_ thin film can be seen in Supplementary Fig. 1a, b. After that, an extra ion-milling process with voltage of 4.5 kV was carried out for 3 min to generate the α- to ε-Fe_2_O_3_ phase transformation. During the ion-milling process, the incident angles of the left and right guns were set as 5° and −5°. The beam currents of the two guns were 54 − 60 μA. The Ar flow was set at 0.12 sccm. The cold figure temperature was 30 °C. Careful TEM investigations revealed that the whole visible area of the film completely transformed into the ε-Fe_2_O_3_ phase, as shown in Supplementary Fig. 1c, d. The PLD-deposited ε-Fe_2_O_3_ thin films were grown on the Nb-doped (0.6 atom %) SrTiO_3_ (111) substrates using a commercial PLD system. During the deposition process, the substrate temperature was set 800 °C. The laser energy was kept at 400 mJ and the oxygen pressure was stabilized at 10 Pa. After the deposition, the samples were cooled steadily to room temperature at 10 °C/min rate. HAADF images were recorded using a 300 kV dual spherical aberration-corrected TEM (Titan G2 60-300, FEI). Optimized STEM parameters were adopted for the HAADF imaging: probe size ~1 Å, probe convergence angle ~ 25 mrad, and collection semi-angle 50–250 mrad. Simulated HAADF images were obtained using the QSTEM package^33^. The macroscopic ferroelectric P-E hysteresis loop of the PLD-deposited ε-Fe_2_O_3_ thin films was measured using a precision TF analyzer 3000 (aixACCT GmbH, Germany) at room temperature and frequency of 300 Hz. The top electrodes were fabricated by depositing circular Au with a diameter of 0.2 mm and a thickness of approximately 100 nm using ion sputtering, while an Nb-doped STO substrate was utilized as the bottom electrode. The PFM measurements were conducted using a Cypher S Asylum Research platform. A conductive tip-coated Ti/Ir (5/20) (ASYELEC.01-R2, Asylum Research) was used to acquire piezoresponse maps and local switching spectra. The magnetic hysteresis loop of the PLD-deposited ε-Fe_2_O_3_ thin films was measured at 300 K using a SQUID system.

### First-principles calculations

First-principles calculations were carried out using the Vienna ab initio simulation package (VASP)^34,35^. The Perdew−Burke−Ernzerhof (PBE) functional^36^ of generalized gradient approximation (GGA) was implemented for all the calculations. The effective Hubbard parameter *U*_eff_ = 3.8 eV (*U*_eff_ = *U* − *J*)^30,37,38^ was used in the GGA + *U* method^39^ for the Fe 3d electrons^40^. An energy cutoff of 500 eV was adopted in the projector augmented wave (PAW) pseudopotential method^41,42^. The structure optimization was performed until all the Hellmann–Feynman forces were less than 10 meV/Å. 4 × 2 × 2 and 4 × 2 × 1 Monkhorst–Pack k-point mesh^43^ were used for the Brillouin zone integration of the unit cell and supercell models, respectively. The relationship between d_FeB-FeC_ and ferroelectric polarization was clarified by first-principles calculations. Based on the HAADF-STEM images of the DWs, the atomic structure models were built in 1 × 1 × 4 surpercells with the lattice parameters obtained from the optimized unit cell of ε-Fe_2_O_3_. The lattice parameters of the bulk obtained in our work are *a* = 5.13 Å, *b* = 8.86 Å and *c* = 9.57 Å, which are consistent with those in the previous work^44^. The polarization at the position of the atom row *i* was calculated by the equation

1 $${{{{{\bf{P}}}}}}^{i}=\frac{e}{{\Omega }_{{{{{\rm{c}}}}}}}\sum {w}_{\alpha }{Z}_{\alpha }^{\ast }{{{{{\bf{u}}}}}}_{\alpha }^{i},$$

where *e*, Ω_c_, *w*_*α*_, *Z*_α_^*^, and **u**_*α*_^*i*^ denote respectively the electron charge, the volume of unit cell, the weight factor, the Born effective charge, and the relative displacement of the *α* ion (Fe and O) in the unit cell at the position of the atom row *i*. Since the changes of bonding across the DWs make the atoms adjacent to the DWs have no exactly corresponding atoms in the bulk, such as Fe_A_, Fe_B_, Fe_C_ and Fe_D_, we use averaged Born effective charges to calculate the polarization near the DWs and ensure the sum of the Born effective charges of all the ions is always zero. Thus spontaneous polarization is calculated to be *P*_s_ = 28 μC/cm^2^ using the averaged Born effective charges *Z*_Fe_* = 4.23 and *Z*_O_* = −2.82 obtained through the berry phase calculation. The *P*_s_ calculated using averaged Born effective charges is relative lager than that calculated using one-to-one Born effective charges in the previous work^41^, which is *P*_s_ = 22.6 μC/cm^2^. The formation energy of the DWs were calculated by

2 $${\Delta E}_{{{{{\rm{DW}}}}}}=\,({E}_{{{{{\rm{tot}}}}}}-{E}_{{{{{\rm{bulk}}}}}})/S,$$

where *E*_tot_ and *E*_bulk_ denote the total energies of the model of domain wall and the bulk with the same number of atoms, and *S* is the area of domain wall in the model.

### Reporting summary

Further information on research design is available in the Nature Portfolio Reporting Summary linked to this article.

### Supplementary information

Supplementary Information

Reporting Summary

## Data Availability

The authors declare that all other relevant data supporting the findings of the study are available in this article and in its Supplementary Information file. Access to our raw data can be obtained from the corresponding author on reasonable request.

## Code availability

The computer code that supports the findings of this study is available from the corresponding authors upon reasonable request.
