# Supplementary material for: Ferroelectric polarization and magnetic structure at domain walls in a multiferroic film
Source: Nat Commun. 2024 Jul 19;15:6099. doi: 10.1038/s41467-024-50431-9 (PMC11271601; doi:10.1038/s41467-024-50431-9)
Supplement: Supplementary file 1 — Supplementary Information [file 41467_2024_50431_MOESM1_ESM.pdf]

Supplementary information for

Ferroelectric polarization and magnetic structure at domain walls in a multiferroic film

Ang Tao[1,2,3,8], Yixiao Jiang[1,3,8], Shanshan Chen[1,2,3,8], Yuqiao Zhang[4,5], Yi Cao[1,2], Tingting Yao[1,3], Chunlin Chen[1,3*], Hengqiang Ye[3] & Xiu-Liang Ma[1,6,7*]

[1]*Shenyang National Laboratory for Materials Science, Institute of Metal Research, Chinese Academy of Sciences, Shenyang 110016, China*

[2]*School of Materials Science and Engineering, University of Science and Technology of China, Shenyang 110016, China*

[3]*Jihua Lab, Foshan 528251, China*

[4]*Institute of Quantum and Sustainable Technology (IQST), School of Chemistry and Chemical Engineering, Jiangsu University, Zhenjiang 212013, Jiangsu, China*

[5]*Foshan (Southern China) Institute for New Materials, Foshan 528200, Guangdong, China*

[6]*Bay Area Center for Electron Microscopy, Songshan Lake Materials Laboratory, Dongguan 523808 Guangdong, China*

[7]*Institute of Physics, Chinese Academy of Sciences, Beijing 100190, China*

[8]these authors contributed equally: Ang Tao, Yixiao Jiang and Shanshan Chen

*Corresponding authors: Chunlin Chen (clchen@imr.ac.cn), Xiu-Liang Ma (xlma@iphy.ac.cn).

This file contains:

Supplementary Note 1

Supplementary Table 1

Supplementary Figures 1-14

Supplementary Note 1. The polarization direction of the ε-Fe_2O_3

The polarization can be calculated by the equation

$$\mathbf{P} = \frac{e}{\Omega_c}\sum w_\alpha Z_\alpha^* \mathbf{u}_\alpha \quad (1)$$

where e, Ω_c, w_α, Z_α^*, and $\mathbf{u}_\alpha$ denote respectively the electron charge, the volume of unit cell, the weight factor, the Born effective charge, and the relative displacement of the α ion compared to the corresponding paraelectric positions. Since the Z_{Fe}^* are positive and the Z_O^* are negative, the direction of the polarization can be confirmed by the relative displacement between the barycenters of Fe and O ions in an ε-Fe_2O_3 unit cell. The positions of the Fe and O ions along c axis in an ε-Fe_2O_3 unit cell as Fig. 1c shown are listed in Supplementary Table 1, based on which the barycenters of Fe and O ions along c axis in an ε-Fe_2O_3 unit cell are calculated to be 0.5437 and 0.5567. Thus the direction of the polarization is along -c.

Supplementary Table 1. Positions of the Fe and O ions along *c* axis in an ε-Fe_2O_3 unit cell

Atom	z/*c*	z/*c*
Fe_B	0.2933	0.7933
Fe_C	0.3043	0.8043
Fe_A	0.0799	0.5799
Fe_D	0.4972	0.9972
O_1	0.4236	0.9236
O_2	0.4273	0.9273
O_3	0.1968	0.6968
O_4	0.1928	0.6928
O_5	0.1678	0.6678
O_6	0.4319	0.9319

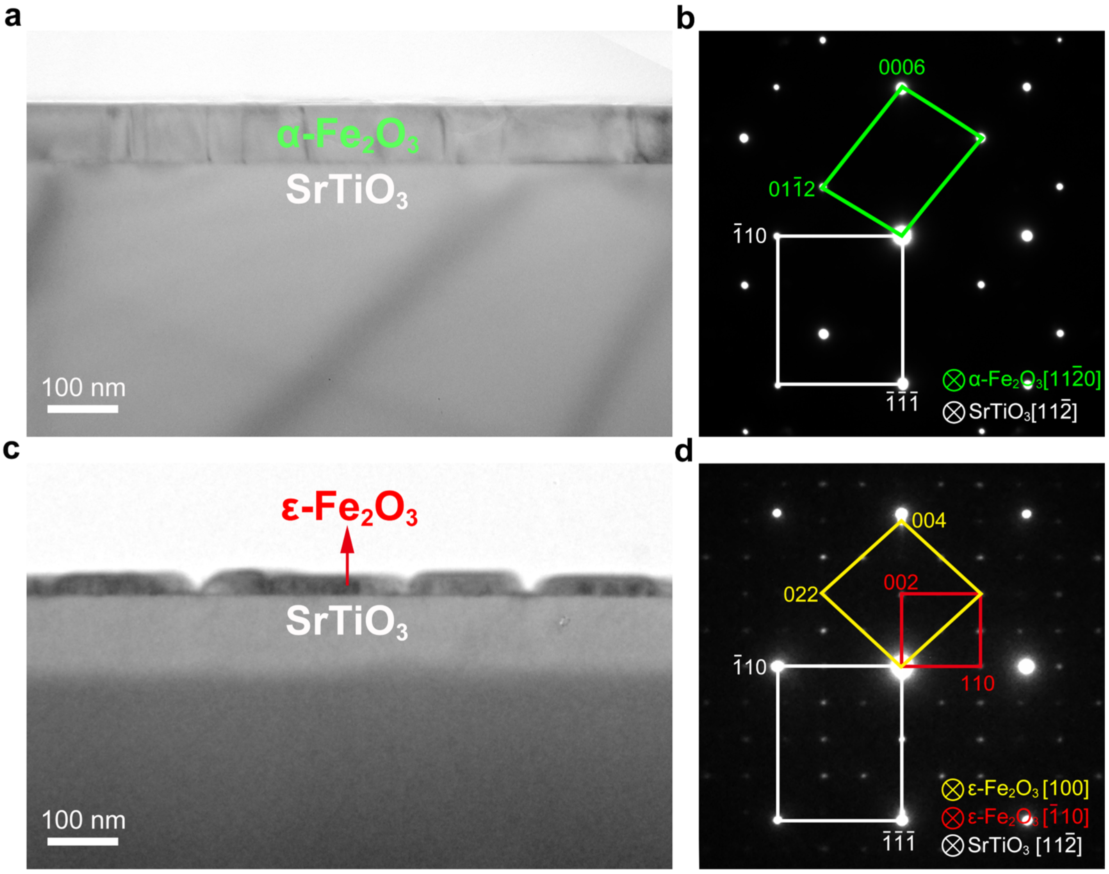

Supplementary Fig.1 | Microstructure of the α-Fe_2O_3 and ε-Fe_2O_3 thin films before and after the 4.5 kV ion-milling process, respectively. (a) Bright-filed TEM image showing the α-Fe_2O_3 film on the $SrTiO_3$ substrate and **(b)** corresponding SAED pattern. **(c)** Bright-filed TEM image showing the transformed ε-Fe_2O_3 film and **(d)** Corresponding SAED pattern. Two types of orientation relationships between film and substrate are formed: $[100]_{\varepsilon}$// $[11\bar{2}]_{sub}$, $[001]_{\varepsilon}$// $[111]_{sub}$ and $[\bar{1}10]_{\varepsilon}$// $[11\bar{2}]_{sub}$, $[001]_{\varepsilon}$// $[111]_{sub}$, respectively. The whole visible area of the film completely transformed into the ε-Fe_2O_3 phase.

.

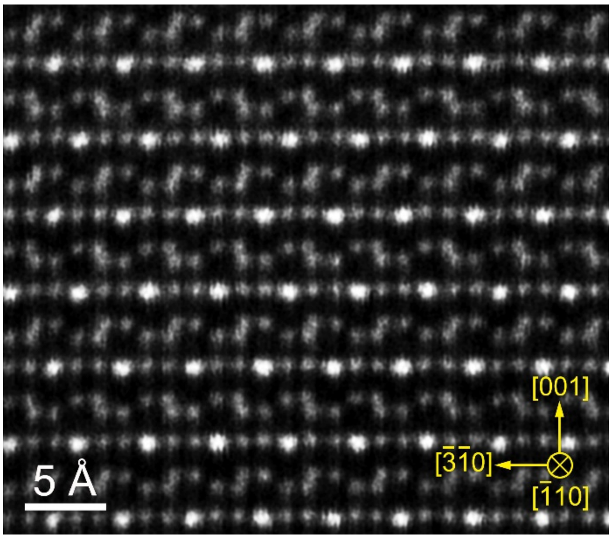

Supplementary Fig. 2 | HAADF-STEM image of the bulk ε-Fe_2O_3 along the [$\bar{1}$10] direction.

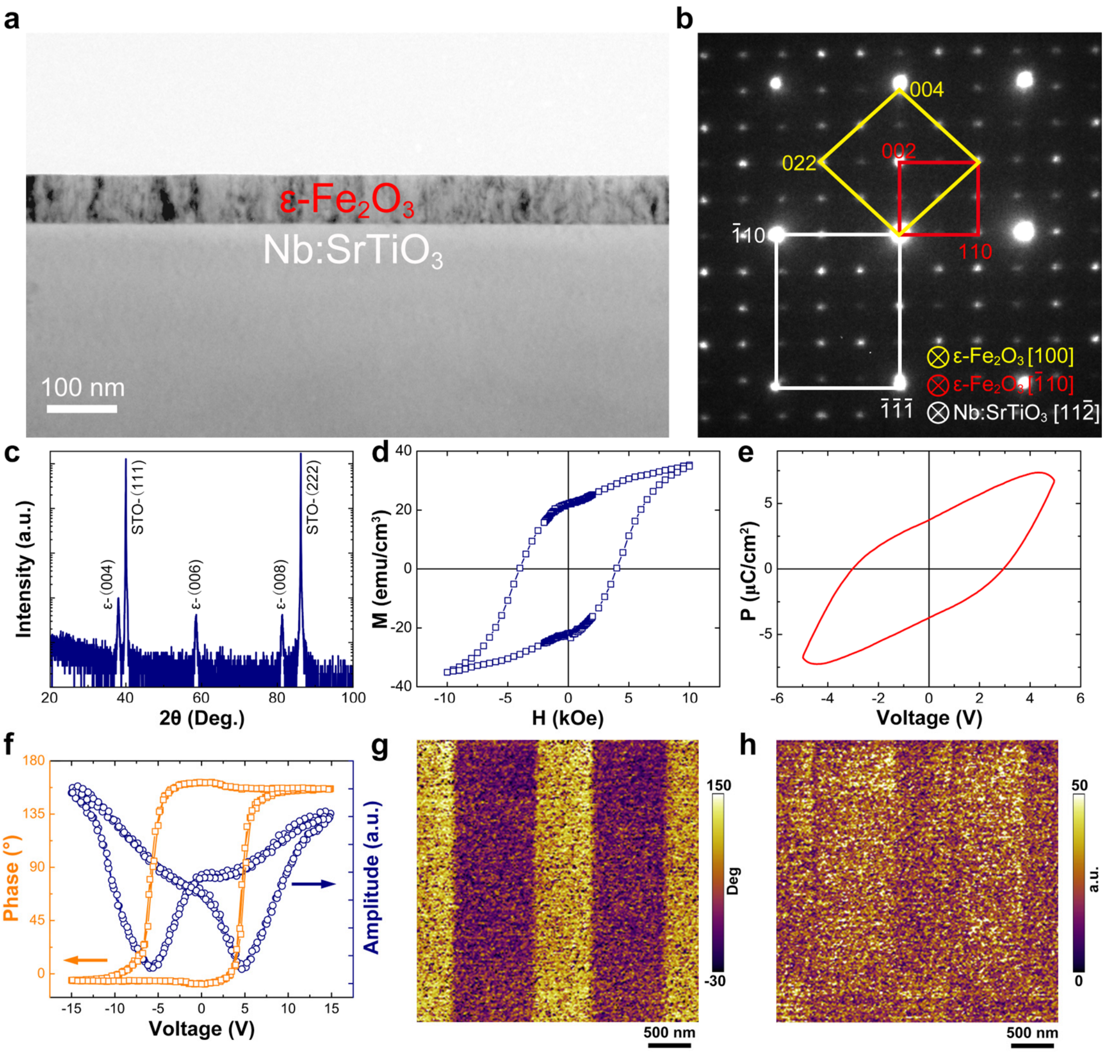

Supplementary Fig. 3 | Ferroelectric and magnetic performance of the PLD-deposited ε-Fe_2O_3 epitaxial film. (a) Bright-filed TEM image showing the PLD deposited ε-Fe_2O_3 epitaxial films on the Nb doped $SrTiO_3$ (111) substrate and **(b)** corresponding SAED pattern. **(c)** XRD pattern showed the pure ε-Fe_2O_3 phase grown epitaxially on the $SrTiO_3$ (111) substrate. **(d)** Magnetization hysteresis loop at room temperature of the ε-Fe_2O_3 film measured with the in-plane magnetic field. **(e)** Ferroelectric hysteresis loop measured at room temperature and hysteresis frequency is 300 Hz. **(f)** Amplitude (navy hollow circles, right axis) and phase (orange hollow squares, left axis) PFM loops of the ε-Fe_2O_3 film. **(g)** Phase and **(h)** amplitude images after poling with +10 V (yellow) and -10 V (dark) regions shown in **(g)**.

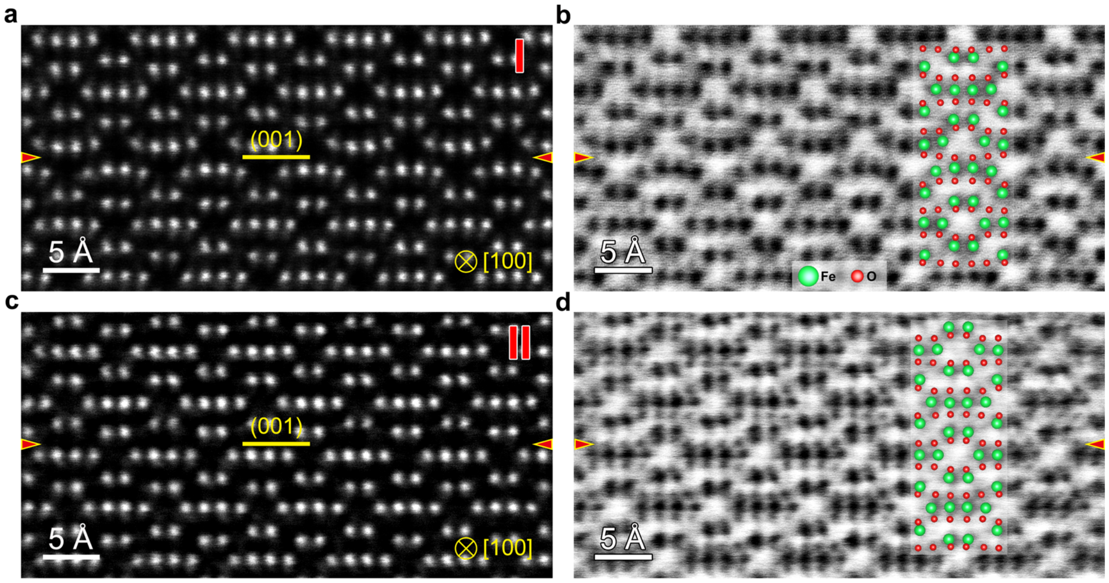

Supplementary Fig. 4 | HAADF and corresponding ABF images of the type I and type II DWs viewed from the [100] projection. (a, c) HAADF STEM images showing the arrangement of Fe atomic columns of the type I and II DWs, respectively. **(b, d)** Corresponding ABF STEM images showing all the atomic columns including O. Atomic models of the type I and type II DWs are inserted in the ABF images.

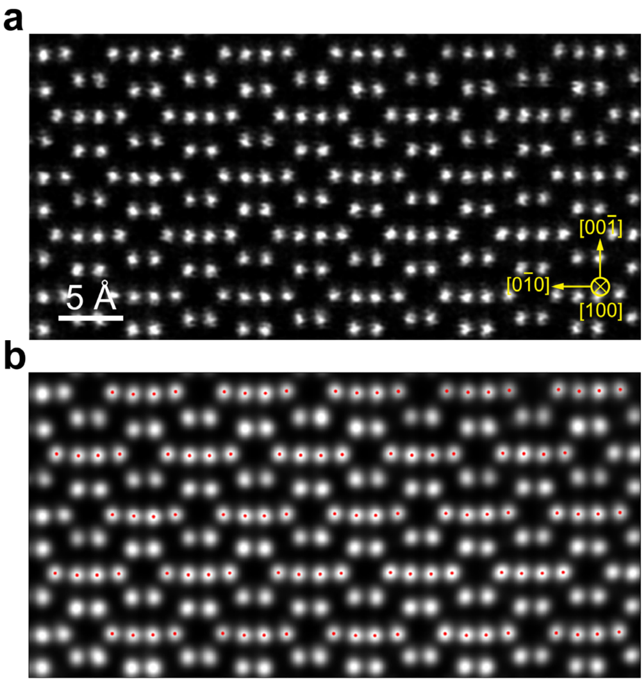

Supplementary Fig. 5 | Determination of the position of atomic column from HAADF-STEM images by using the CalAtom Software. (a) Raw HAADF image of the bulk ε-Fe_2O_3 phase along the [100] direction. **(b)** Corresponding Guess-filtered image and the center position of Fe_B and Fe_C columns (red dots).

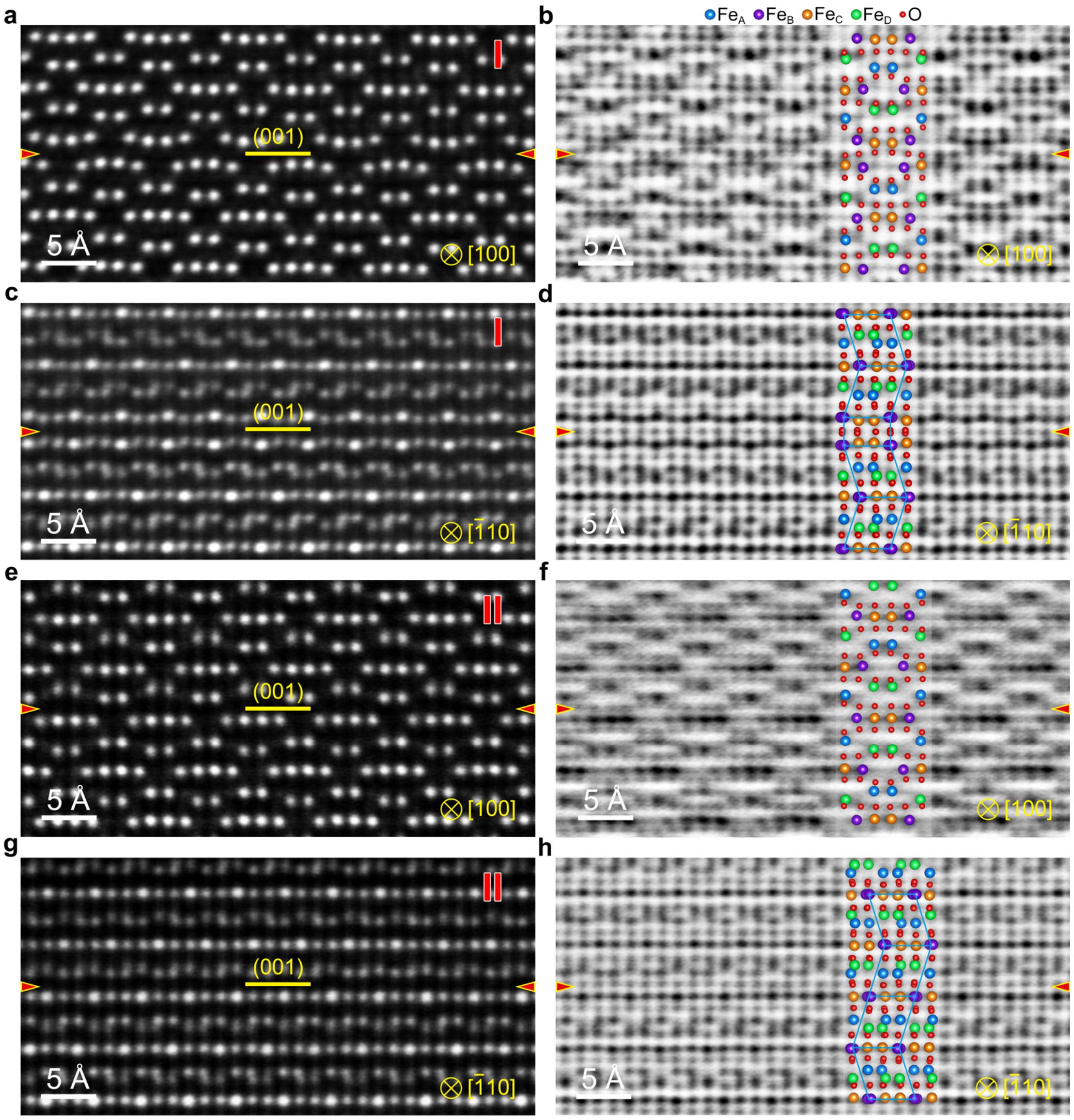

Supplementary Fig. 6 | HAADF and ABF images of the type I and type II DWs viewed from the [100] and [$\bar{1}$10] projection in the PLD-deposited ε-Fe_2O_3 thin films. **(a, c)** HAADF STEM and **(b, d)** corresponding ABF STEM images showing the atomic structures of the type I DW viewing from the [100] and [$\bar{1}$10] projection, respectively. **(e, g)** HAADF STEM and **(f, h)** corresponding ABF STEM images showing the atomic structures of the type II DW viewing from the [100] and [$\bar{1}$10] projection, respectively. Atomic models of the type I and type II DWs are inserted in the ABF images. The type I and type II DWs in the PLD-deposited ε-Fe_2O_3 thin films have the same atomic structures as those in the irradiation-induced ε-Fe_2O_3 thin films.

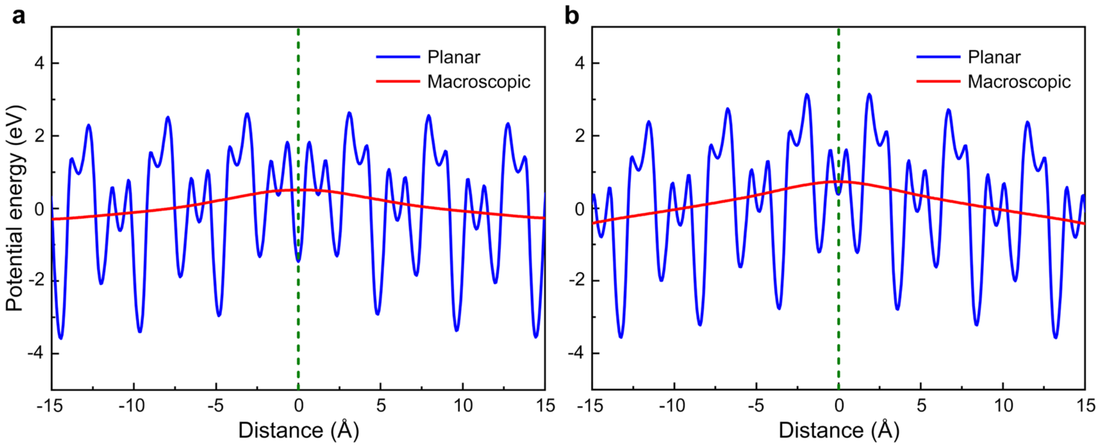

Supplementary Fig. 7 | Potential energy distribution along *c* axis across the two ε-Fe_2O_3 DWs. (a) Potential energy distribution along *c* axis across the type I DW. (**b)** Potential energy distribution along *c* axis across the type II DW. The blue lines denote the planar averaged potential energy, red lines denote the macroscopic averaged potential energy and green dashed lines denote the locations of DWs.

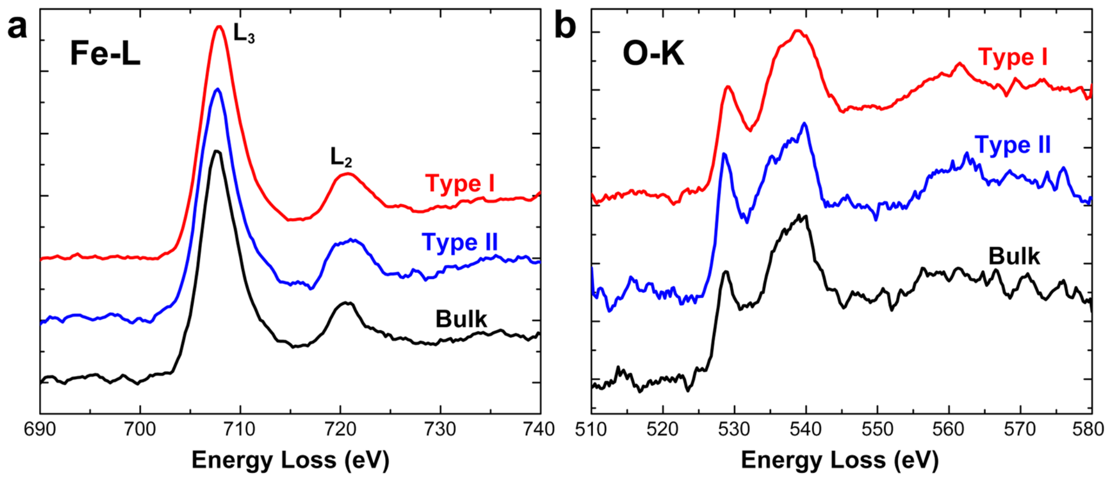

Supplementary Fig. 8 | Fe-L edges and O-K edges obtained from the ε-Fe_2O_3 DWs and the bulk. **(a)** Fe-$L_{2,3}$ and **(b)** O-K edges obtained from the two types of DWs and the bulk, respectively. The Fe-$L_{2,3}$ and O-K edges obtained from the type I and type II DWs are similar with those from the bulk, indicating that the valence states of Fe ions at the DWs and the bulk are 3+.

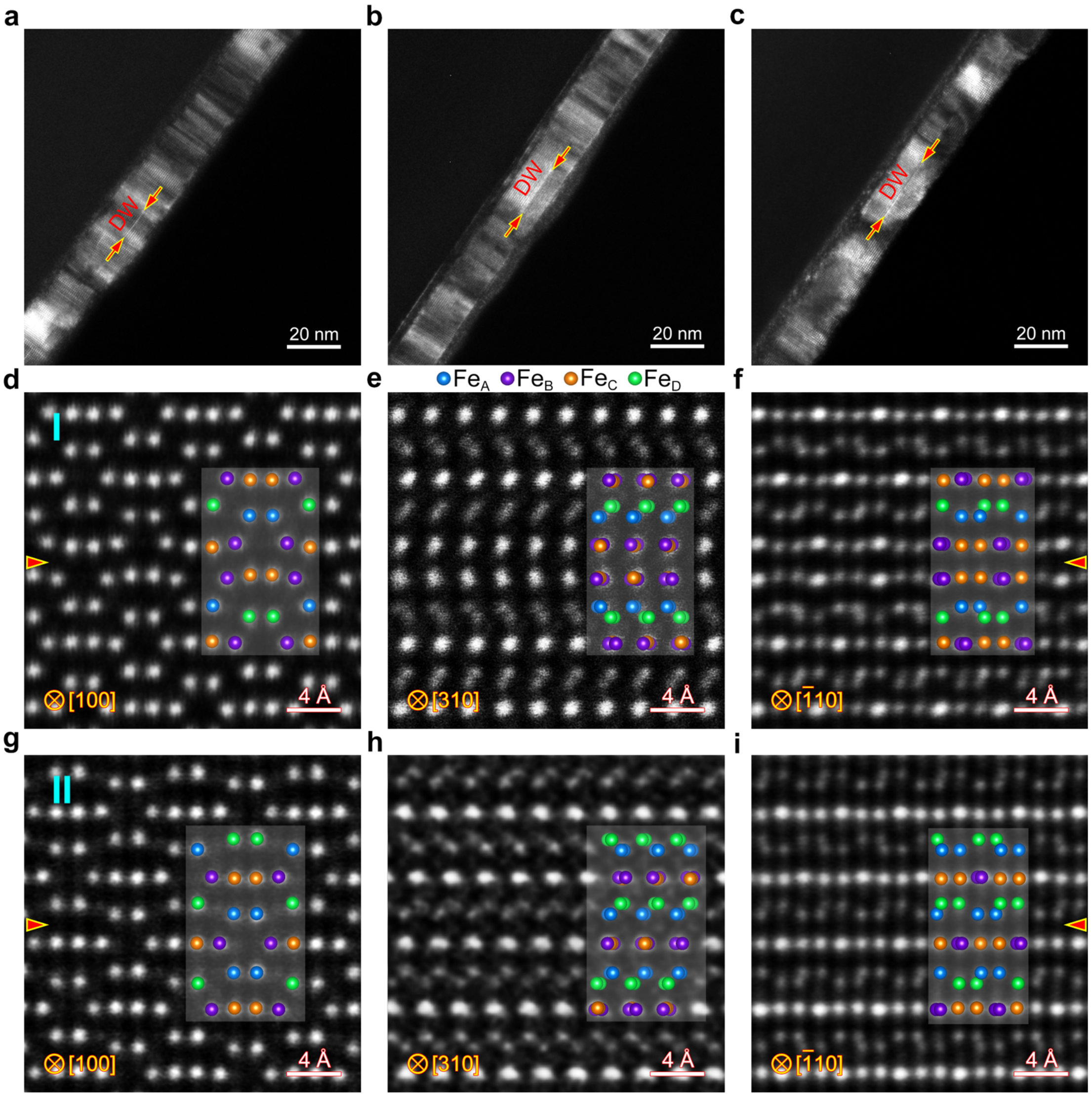

Supplementary Fig. 9 | Microstructures images of the ε-Fe_2O_3 DWs. (a-c) Typical low-mag weak beam dark field TEM images show the DWs which behave weak thin lines and the contrasts remain consistent on both sides of the DWs. The DWs are marked as red arrows. **(d-e)** Atomic-resolution HAADF images of type I DW viewing from representative [100], [310] and [$\bar{1}$10] projections, respectively. **(g-i)** Atomic-resolution HAADF images of type II DW viewing from representative [100], [310] and [$\bar{1}$10] projections, respectively. The atomic models of the DWs overlap on the HAADF images for easy understanding of the atomic structure of the DWs. The DWs are marked as red arrows in **(d-i)**.

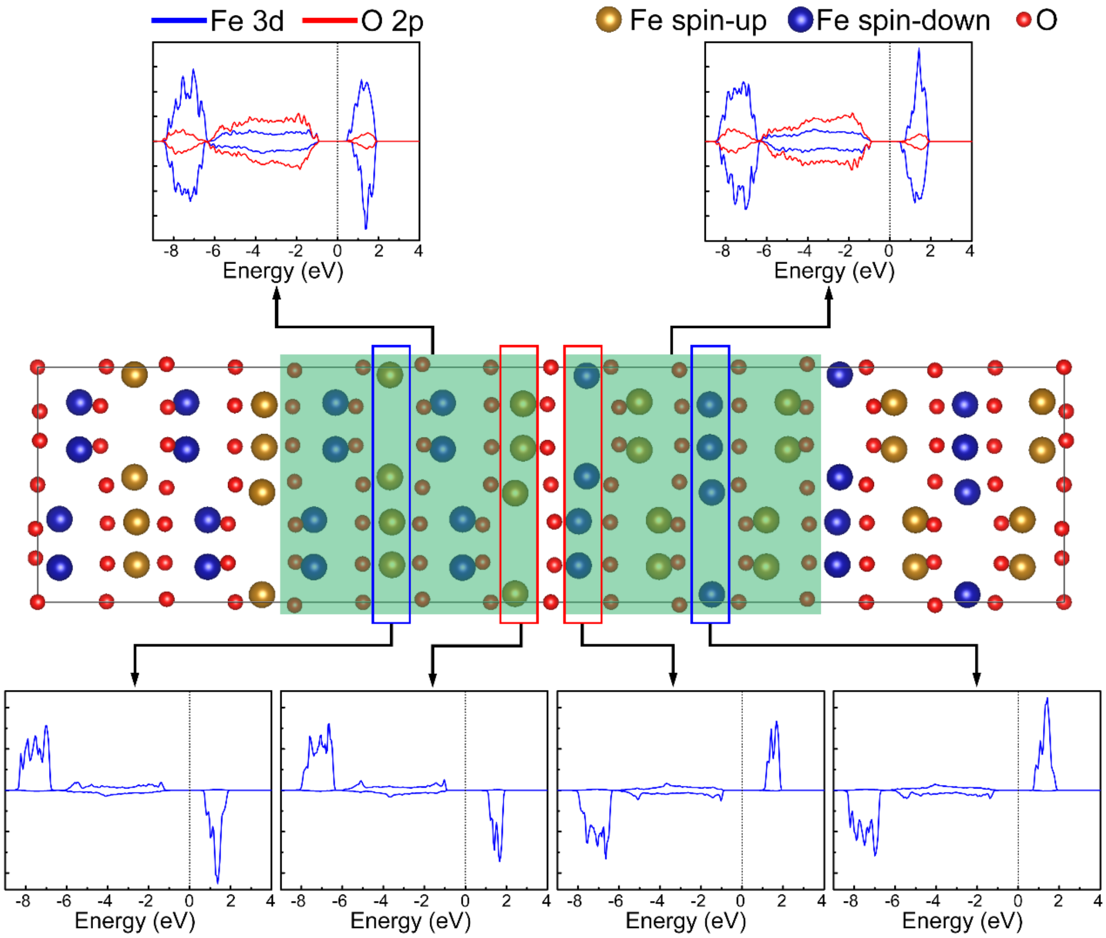

Supplementary Fig. 10 | Spin-polarized local density of states (LDOS) across the type I DW. The upper two figures show the LDOS of the two domains across the type I DW structure. The bottom four figures show the Fe LDOS of the Fe layers near the DW. The net magnetic moments of the two domains adjacent to the type I DW are antiparallel, revealing the AFM coupling nature across the DW.

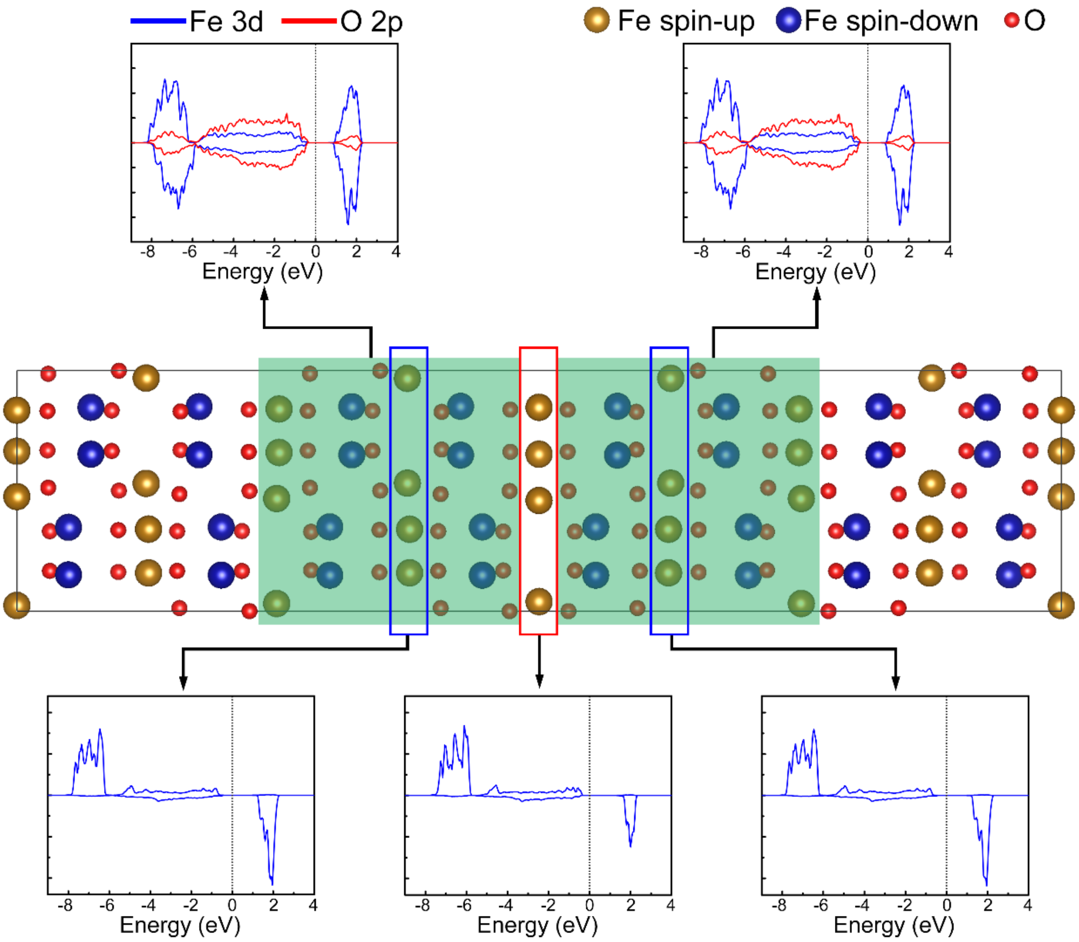

Supplementary Fig. 11 | Spin-polarized LDOS across the type II DW. The upper two figures show the LDOS of the two domains across the type II DW structure. The bottom three figures show the Fe LDOS of the Fe layers near the DW. The net magnetic moments of the two domains adjacent to the type II DW are parallel, revealing the FM coupling nature across the DW.

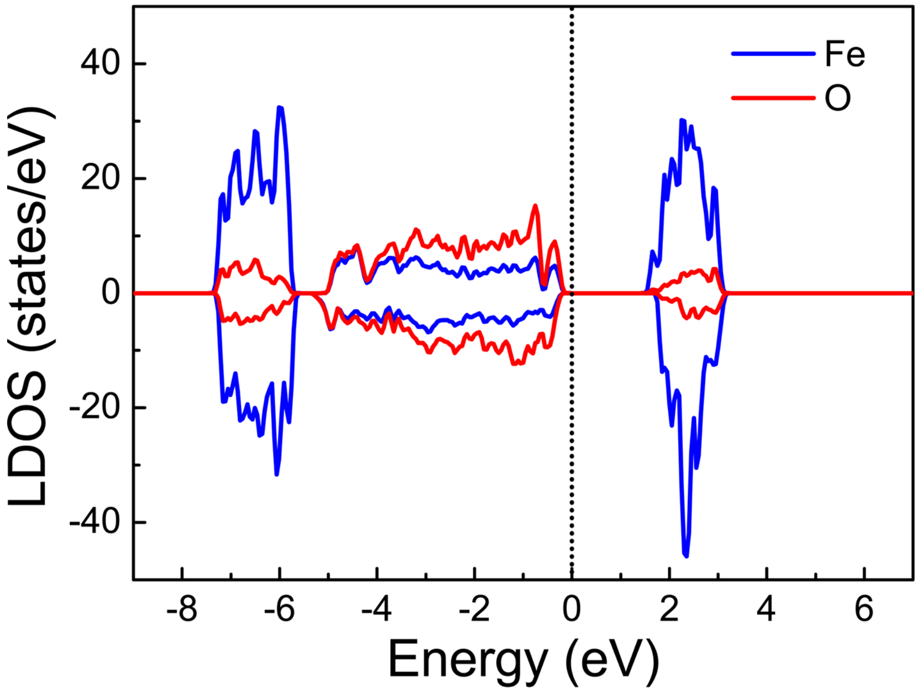

Supplementary Fig. 12 | Spin-polarized local density of states (LDOS) of the bulk ε-Fe_2O_3.

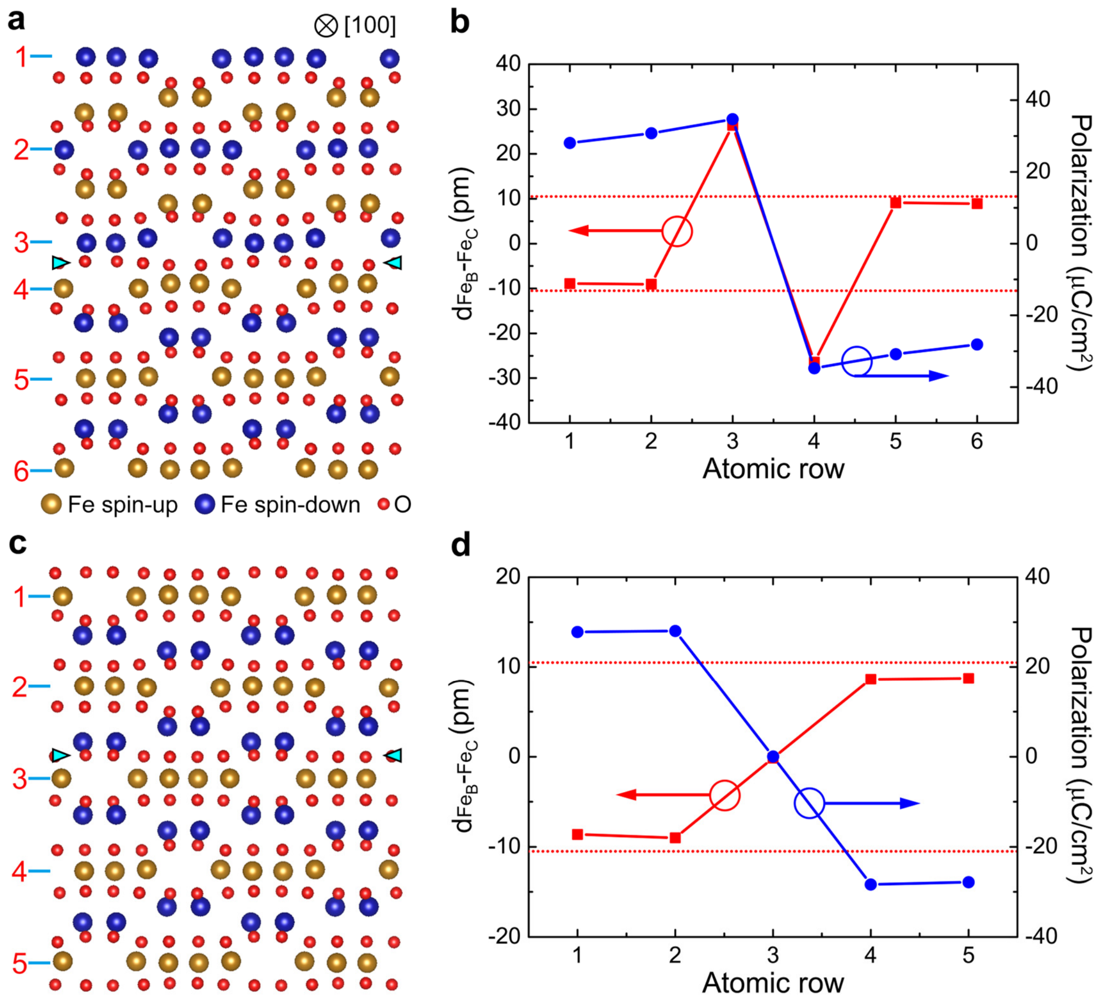

Supplementary Fig. 13 | Ferroelectric polarization in two types of head-to-head ε-Fe_2O_3 DWs calculated by DFT. (a) Atomic models of the type I head-to-head DW. The DWs are denoted by cyan arrows. **(b)** Calculated $d_{FeB-FeC}$ and ferroelectric polarization near the type I head-to-head DW. **(c)** Atomic models of the type II head-to-head DW. **(d)** Calculated $d_{FeB-FeC}$ and ferroelectric polarization near the type II head-to-head DW. The red dotted lines indicate the $d_{FeB-FeC}$ value of the bulk ε-Fe_2O_3 in **(b, d)**.

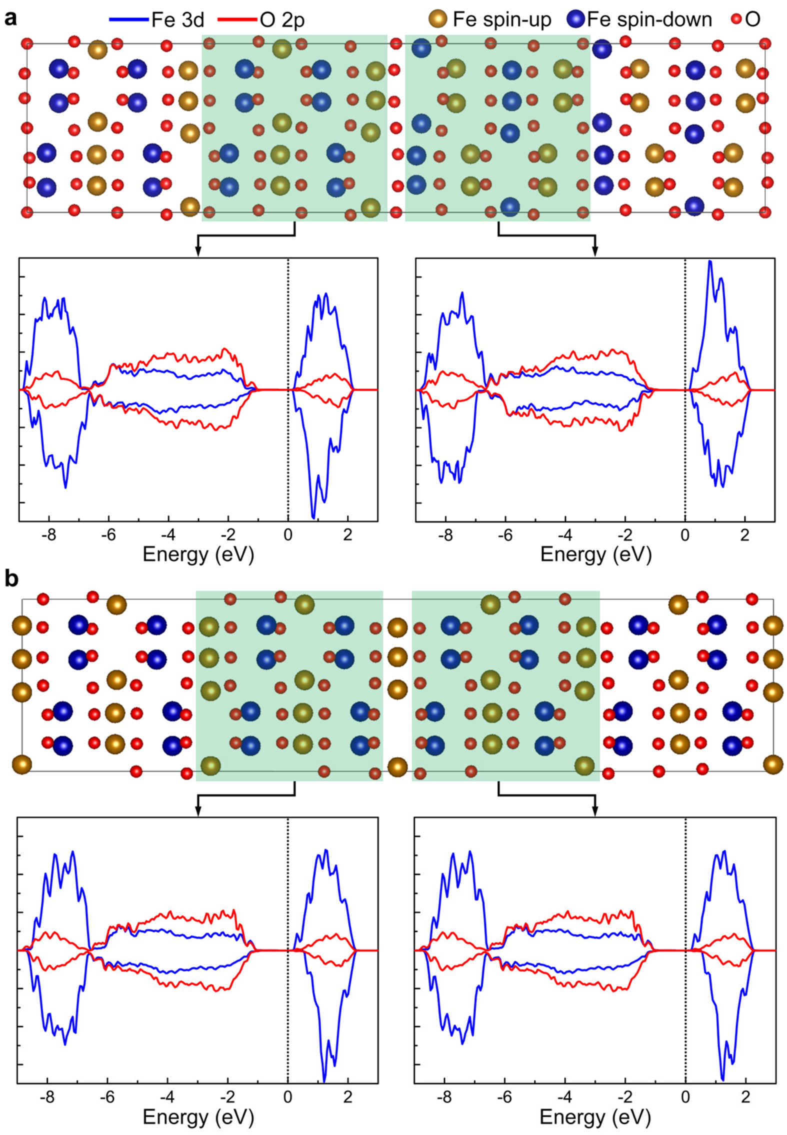

Supplementary Fig. 14 | Spin-polarized local density of states (LDOS) across the type I and type II head-to-head DWs. The E_F is denoted by the dashed lines. (**a**) LDOS across the type I head-to-head DW. The net magnetic moments of the two conjugated domains for the type I DW are antiparallel, revealing the AFM coupling nature across the DW. (**b**) LDOS across the type II head-to-head DW. The net magnetic moments of the two conjugated domains for the type II DW are parallel, indicating the FM coupling nature across the DW.
