# Supplementary material for: Ferroelectric polarization and magnetic structure at domain walls in a multiferroic film
Source: Nat Commun. 2024 Jul 19;15:6099. doi: 10.1038/s41467-024-50431-9 (PMC11271601; doi:10.1038/s41467-024-50431-9)
Supplement: Supplementary file 2 — Reporting Summary [file 41467_2024_50431_MOESM2_ESM.pdf]

nature research

Corresponding author(s): Chunlin Chen, Xiu-Liang Ma

Lasing Reporting Summary

Nature Research wishes to improve the reproducibility of the work that we publish. This form is intended for publication with all accepted papers reporting claims of lasing and provides structure for consistency and transparency in reporting. Some list items might not apply to an individual manuscript, but all fields must be completed for clarity.

For further information on Nature Research policies, including our data availability policy, see Authors & Referees.

• Experimental design

Please check: are the following details reported in the manuscript?

1. Threshold

Plots of device output power versus pump power over a wide range of values indicating a clear threshold
- [ ] Yes
- [x] No

The experimental methods section does not include this information.

2. Linewidth narrowing

Plots of spectral power density for the emission at pump powers below, around, and above the lasing threshold, indicating a clear linewidth narrowing at threshold
- [ ] Yes
- [x] No

The experimental methods section does not include this information.

Resolution of the spectrometer used to make spectral measurements
- [ ] Yes
- [x] No

The experimental methods section does not include this information.

3. Coherent emission

Measurements of the coherence and/or polarization of the emission
- [ ] Yes
- [x] No

The experimental methods section does not include this information.

4. Beam spatial profile

Image and/or measurement of the spatial shape and profile of the emission, showing a well-defined beam above threshold
- [ ] Yes
- [x] No

The experimental methods section does not include this information.

5. Operating conditions

Description of the laser and pumping conditions
Continuous-wave, pulsed, temperature of operation
- [x] Yes
- [ ] No

The relevant informations can be found in methods section

Threshold values provided as density values (e.g. W cm^{-2} or J cm^{-2}) taking into account the area of the device
- [x] Yes
- [ ] No

The relevant informations can be found in methods section

6. Alternative explanations

Reasoning as to why alternative explanations have been ruled out as responsible for the emission characteristics
e.g. amplified spontaneous, directional scattering; modification of fluorescence spectrum by the cavity
- [ ] Yes
- [x] No

The experimental methods section does not include this information.

7. Theoretical analysis

Theoretical analysis that ensures that the experimental values measured are realistic and reasonable
e.g. laser threshold, linewidth, cavity gain-loss, efficiency
- [ ] Yes
- [x] No

The experimental methods section does not include this information.

8. Statistics

Number of devices fabricated and tested
- [ ] Yes
- [x] No

The experimental methods section does not include this information.

Statistical analysis of the device performance and lifetime (time to failure)
- [ ] Yes
- [x] No

The experimental methods section does not include this information.
